# Haematopoietic stem cells: entropic landscapes of differentiation

**DOI:** 10.1098/rsfs.2018.0040

**Published:** 2018-10-19

**Authors:** K. Wiesner, J. Teles, M. Hartnor, C. Peterson

**Affiliations:** 1 School of Mathematics, University of Bristol, Bristol BS8 1TW, UK; 2 Computational Biology and Biological Physics, Department of Astronomy and Theoretical Physics, Lund University, Lund 223 62, Sweden; 3 Sainsbury Laboratory, University of Cambridge, Cambridge CB2 1LR, UK

**Keywords:** stem cell differentiation, Shannon information theory, entropy

## Abstract

The metaphor of a potential epigenetic differentiation landscape broadly suggests that during differentiation a stem cell approaches a stable equilibrium state from a higher free energy towards a stable equilibrium state which represents the final cell type. It has been conjectured that there is an analogy to the concept of entropy in statistical mechanics. In this context, in the undifferentiated state, the entropy would be large since fewer constraints exist on the gene expression programmes of the cell. As differentiation progresses, gene expression programmes become more and more constrained and thus the entropy would be expected to decrease. In order to assess these predictions, we compute the Shannon entropy for time-resolved single-cell gene expression data in two different experimental set-ups of haematopoietic differentiation. We find that the behaviour of this entropy measure is in contrast to these predictions. In particular, we find that the Shannon entropy is not a decreasing function of developmental pseudo-time but instead it increases towards the time point of commitment before decreasing again. This behaviour is consistent with an increase in gene expression disorder observed in populations sampled at the time point of commitment. Single cells in these populations exhibit different combinations of regulator activity that suggest the presence of multiple configurations of a potential differentiation network as a result of multiple entry points into the committed state.

## 1. Introduction

The programmes governing the function and fate of cells are to a large extent driven by the coordinated activity of transcription factors forming complex and dynamic gene regulatory networks (GRNs). The activities of transcription factors and other genes involved in cell fate decisions can be measured by a number of different gene expression quantification experiments. Until recently, and due to technical limitations, for a given cell type such experiments had to be done on an ensemble of many cells and, hence, gene expression quantifications represented the average over a given population. This averaging effect hampered the analysis of finer regulatory mechanisms at the single-cell level, the fundamental unit for any fate decision process. More recently, a number of novel technologies have facilitated gene expression measurements for individual cells, thereby opening up the possibility of quantifying heterogeneity among cells of a given population and between related populations (for a review, see, for example, [1]). Such heterogeneity could originate from extrinsic factors, such as cell-to-cell signalling and surrounding temperature and pressure, but also from the intrinsic noise generated by having few copies of molecules involved in transcription and translation. Whether intrinsic noise is simply a result of the stochastic nature of any cellular process or it actually plays a mechanistic role in cellular decision-making processes during differentiation is currently the object of intense study.

Entropy in statistical mechanics is a measure of disorder in the macrostate of a system. The more different microstates are visited the higher the entropy. Mathematically, the statistical mechanical entropy is equivalent to the information-theoretic Shannon entropy, where the latter measures the amount of randomness in a probability distribution [2]. Hence, the Shannon entropy of a probability distribution over gene expression levels in a cell population measures the amount of randomness or heterogeneity in its gene expression patterns. Therefore, estimating the Shannon entropy of a cell population might yield insights into the role of gene expression heterogeneity, which would be of particular interest in a context of state transitions such as cellular differentiation.

With the upsurge of studies of stem cell commitment processes during the last decade the subject of heterogeneity is of particular interest. Since stem cells and progenitors host the genetic programme potential for all mature cell types they can give rise to, one would naively expect them to be strongly disordered in terms of gene expression patterns compared with the mature cells they originate. Expressing order or disorder as a lack thereof by means of entropy could be a way forward in monitoring commitment of stem cells, and differentiation towards mature cells.

**Figure 1.**
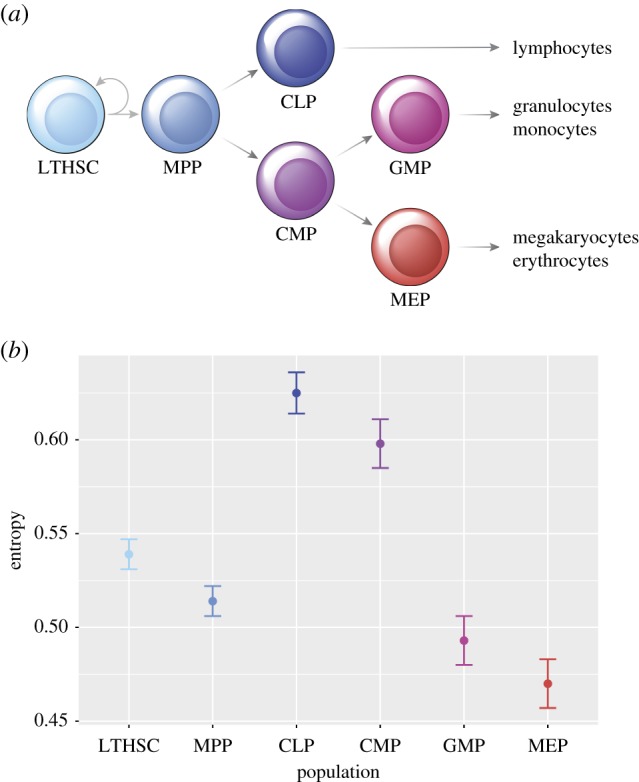
Binary Shannon entropies during haematopoietic differentiation. (*a*) Depiction of a haematopoietic stem cell differentiation tree. For each of the cellular populations, we used single-cell gene expression for a number of relevant genes as quantified in [3]. LTHSC, long-term haematopoietic stem cell; MPP, multipotent progenitor; CMP, common myeloid progenitor; CLP, common lymphoid progenitor; GMP, granulocyte–monocyte progenitor; MEP, megakaryocyte–erythroid progenitor. (*b*) Binary Shannon entropy estimates based on single-cell expressions of all genes for each population in (*a*), with standard error obtained with the jackknife method (see text for details; the values are given in table 1). A significant increase in entropy can be observed immediately after the first branching point, between MPP and CLP/CMP.

We have therefore explored such scenarios of stem cell commitment and differentiation for two haematopoietic differentiation systems. (i) The first system [3] consists of long-term haematopoietic stem cells (LTHSCs) which differentiate into multipotent progenitors (MPPs) before bifurcating into common myeloid progenitors (CMPs) or common lymphoid progenitors (CLPs), as illustrated in figure 1*a*. In this first system, we are interested in quantifying the entropy while the system moves from less differentiated to more differentiated compartments and, in particular, in assessing how the entropy behaves before and after the first major branching point. (ii) The second system is an example of haematopoietic differentiation at a more fine-grained resolution. We use gene expression data immediately before and after an erythroid commitment decision [4] in the factor-dependent multipotent haematopoietic cell line erythroid myeloid lymphoid (EML). As in the first system, we are interested in assessing how entropy values change from a less to a more constrained differentiation state, across the point where an irreversible decision has been made.

**Table 1.** Normalized binary Shannon entropies during haematopoietic differentiation for pairs of genes (*H*(*P*_12_)) and single genes (*H*(*P*)) including standard deviation, for a number of relevant genes as quantified in [3]. The values *H*(*P*) are plotted in figure 1.

	*H*(*P*_12_)	*H*(*P*)
LTHSCs	0.534 ± 0.001	0.539 ± 0.008
MPPs	0.508 ± 0.001	0.514 ± 0.008
CLPs	0.605 ± 0.001	0.625 ± 0.011
CMPs	0.576 ± 0.001	0.598 ± 0.013
GMPs	0.476 ± 0.001	0.493 ± 0.013
MEPs	0.457 ± 0.001	0.470 ± 0.013

## 2. Single-cell gene expression data

For this study, we considered two sets of previously published single-cell quantitative reverse transcription polymerase chain reaction (RT-qPCR) data that included candidate genes known to be involved at different stages of haematopoietic differentiation. From Guo *et al.* [3], we analysed the data from 179 regulators that included lineage-specific transcription factors, epigenetic modifiers and cell-cycle regulators. The expression of these genes was quantified in a total of 191 cells from different stem and progenitor cell populations: LTHSCs, MPPs, CLPs, CMPs, granulocyte–monocyte progenitors (GMPs) and megakaryocyte–erythroid progenitors (MEPs). For each gene, expression is defined as log_2_ expression above the system background *Ct* of 28 (i.e. 28 minus the measured raw *Ct*). *Ct* values higher than 28 were transformed to 28 and defined as being 0 (no measurable gene expression). For more experimental details on population sorting, the PCR protocol and gene filtering/normalization we refer to the original paper [3]. From Pina *et al.* [4], we analysed single-cell gene expression data from different subpopulations of the multipotent haematopoietic cell line EML. More specifically, we focused on RT-qPCR data for 17 genes measured in 319 self-renewing (SR), 109 erythroid-committed (CP) and 83 erythroid-differentiated (Ediff) cells. Through clustering and multivariate methods, the CP population was further subdivided into two compartments, CP1 and CP2, as described in Teles *et al.* [5]. CP1 and CP2 have been inferred to be early and late committed cells, respectively, given the similarity of their gene expression profiles to the SR (in the case of CP1) or Ediff (in the case of CP2) populations. For all genes, expression was originally defined as Δ*Ct* for each gene to the reference gene (Atp5a1) and linearly transformed to ln(2^30^ – Δ*Ct*), where 30 is the experimental detection limit. For more information on culture conditions, cell sorting and gene filtering/normalization we refer to [4].

## 3. Entropy estimation

The standard Shannon entropy is a function of a discrete probability distribution while gene expression, in general, is measured on a continuous scale. Hence, the data need to be discretized for the entropy to be measured. The alternative is to estimate the generalized Shannon entropy for continuous distributions (for example [2]). However, both definition and estimation of continuous Shannon entropy are afflicted with problems, such as requiring large data and potentially returning negative values. We, therefore, do not consider the continuous Shannon entropy any further here, but we will offer some insights into its use in the context of gene expression data in a forthcoming publication.

In discretizing continuous gene expression data into bins, the decision of how many bins to use is a difficult one when there is no obvious and biologically justified separation between expression levels. Hence, in this study, only two obviously separate levels are distinguished between: the zero expression level and the greater-than-zero expression level. From this, the binary Shannon entropy (equation (3.1)) is estimated. The Shannon entropy of a binary probability distribution *P* over two events (representing the two bins), each with probability *p*_0_ and *p*_1_, respectively, is defined as3.1
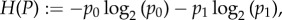
where 0 log 0 := 0. The Shannon entropy is symmetric in the probabilities of the two events, it is zero whenever either *p*_0_ = 0 or *p*_1_ = 0, and it is maximal when 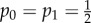, in which case *H*(*P*) = 1.

The Shannon entropy for a joint probability distribution is defined in a similar way. Let *P*_12_ be a joint distribution over two binary events, with respective probabilities *p*_00_, *p*_01_, *p*_10_ and *p*_11_. Then the Shannon entropy over this joint distribution is defined as3.2
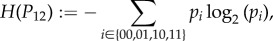
with 0 log 0 := 0 as before.

The entropies of the gene expression data in this study were estimated using the maximum-likelihood method. It is known that for cases of few bins and many data points this estimator is optimal (e.g. [6, p. 1470]). The results were compared with those obtained with other estimators such as the non-parametric James–Stein-type shrinkage estimator, developed by Hausser & Strimmer [6], and the Miller Meadow estimator. No qualitative difference was observed. The minor observed quantitative differences were due to a systematic overcorrection in the Miller Meadow estimator which lead to single entropies larger than 1, and due to a mismatch between single entropies (*H*(*P*)) and self-joint entropies (*H*(*P*_11_)) in the James–Stein-type estimator. All estimators, together with other entropy estimators, were computed using the R package ‘entropy’ [7].

Entropy is not the only measure of randomness or variation of a random variable. An obvious one to compare it with is the variance. In the case of a binary random variable, there is a straightforward mathematical relation between the variance and the entropy. Using the same notation as in equation (3.1), the variance of a binary random variable is given by3.3
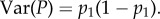
The variance and the entropy of a binary probability distribution both peak at 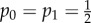 and are equal to zero for *p*_0_ = 0 or *p*_0_ = 1. Thus, the variance computed for the same dataset will show the same qualitative behaviour as the entropy. We computed the sample variance for both gene expression data sets (not included here) and found this mathematical prediction confirmed.

The true strength of the Shannon entropy over other statistical measures of randomness is both that it can be generalized to a set of *n* correlated random variables and that it is an entry point to a whole set of information-theoretic tools which quantify randomness of and correlations between any number of variables. Less relevant here but still worth noting is that the Shannon entropy is applicable to data which are non-numeric, such as DNA sequences, molecular configurations or written text. Furthermore, as mentioned in the beginning, the Shannon entropy is proportional to the statistical mechanical Gibbs entropy (although the debate on the interpretation of this mathematical fact is still ongoing [8]). Hence, the Shannon entropy can be used directly in discussions of a potential epigenetic differentiation landscape imposing statistical mechanical constraints on genetic development through the laws of thermodynamics.

### 3.1. Standard error of entropy estimates

To obtain the standard error (the root mean squared error) of the entropy estimates, the non-parametric jackknife method was used [9]. There are many comprehensive expositions of this method, e.g. [10,11]. We briefly summarize it here: for a set of *n* samples of a random variable (r.v.), an estimator 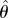 of the r.v. (such as the mean, the variance or the entropy) is computed *n* times, each time with one of the data points being removed. Call this estimate 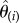, where the 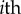 data point was removed. Efron showed [9] that the standard error of the estimate is given by3.4
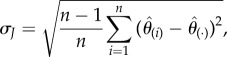
where 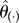 is the average of the estimates,3.5
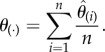


## 4. Results

### 4.1. Long-term haematopoietic stem cell differentiation

We estimated the binary Shannon entropy for all cell populations as defined by surface markers of the haematopoietic differentiation tree (figure 1*a*) described in [3] from which the gene expression data are also taken. The results are shown in figure 1. Contrary to what has been conjectured and to what could intuitively *a priori* be expected, entropy was not found to be a continuously decreasing function along the differentiation pathway (figure 1*a*). Instead, we observed that entropy slightly decreases from the LTHSC stage to the MPP stage and shows a significant increase between the MPP stage and both the CLP and the CMP stages, before decreasing again sharply between the CMP and both the GMP and the MEP stages.

We have also computed the joint binary Shannon entropy for all pairs of genes, shown in table 1. The observed trend is the same as for the marginal (single gene) entropy: a slight decrease from the LTHSC stage to the MPP stage, a significant increase between the MPP and both the CLP and the CMP stage, and a sharp decrease again between the CMP and both the GMP and the MEP stages. The slightly lower values of (normalized) *H*(*P*_12_) compared with *H*(*P*) indicate that there are correlations in the gene expression data. Note that *P* is a marginal distribution over single gene expressions while *P*_12_ is the joint distribution. In general, for a joint probability distribution *P*_12_ and its marginal *P*_1_ and *P*_2_ (in our case the two marginals are equal due to symmetry), the difference between the two (normalized) entropies, 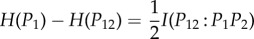, is half the mutual information 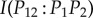, where *P*_1_*P*_2_ is the product distribution. The mutual information is a measure of correlation on the joint distribution [2]. Such correlations may suggest some level of coordination in the expression programmes, which could potentially decrease the level of entropy when considering two genes together when compared with the entropies of single genes separately.

### 4.2. EML cell line erythroid commitment

**Figure 2.**
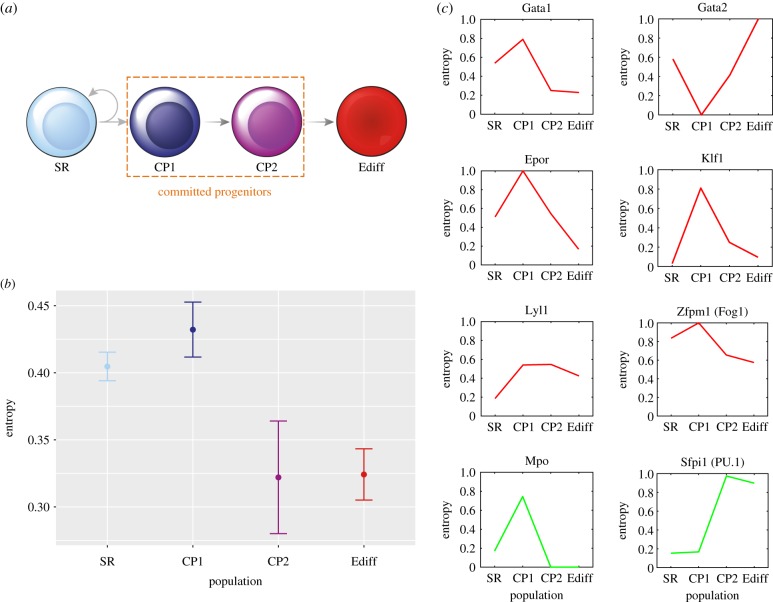
Binary Shannon entropies of the EML cell line. (*a*) Depiction of subpopulations of the EML cell line allowing the capture of states immediately before (SR, self-renewing cells) and after (CP, committed progenitors) commitment. For each population, we used single-cell gene expression quantification for a number of candidate genes as measured in [4]. CP1 and CP2 are, respectively, early and late committed progenitors; Ediff, erythroid-differentiated cells. (*b*) Binary Shannon entropy estimates for all genes in each population in (*a*), with standard error obtained with the jackknife method (see text for details). Entropy values increase immediately after the commitment boundary, in the transition between SR and CP1, decreasing again from CP1 to CP2 and Ediff. (*c*) Binary Shannon entropy estimates for known genes of interest in erythroid (red) and myeloid (green) differentiation (error bars omitted for simplicity). For the remaining genes in the dataset, please see the electronic supplementary material, figure S1.

To further investigate entropy dynamics during differentiation, we estimated binary entropies for subpopulations of the EML cell line immediately before and after erythroid commitment, from SR to CP populations [4,5] (figure 2*a*). As can be seen in figure 2*b*, the entropy values are highest immediately after the decision point, similar to what we observed for the MPP to CMP/CLP transition. Entropy increases from SR to CP1 and decreases again from CP1 to CP2 and from CP2 to Ediff, the late commitment and terminally differentiated populations, respectively.

As previously described by the authors of [4,5], CP1 cells show heterogeneity in the expression of known regulators of the erythroid lineage such as Gata1 and Klf1. This observation is consistent with the notion that commitment can be effected even in the absence of the so-called master regulators, and that multiple network configurations can coexist immediately after commitment, subsequently consolidating and becoming more homogeneous in the population as cells differentiate. We tried to further explore this scenario by analysing the single-gene entropy behaviours for genes involved in erythroid differentiation before and after commitment (i.e. in SR versus CP1 populations). As can be seen in figure 2*c*, Gata1, Zfpm1, Klf1, Epor and Lyl1 all show an increase in entropy from SR to CP1, subsequently decreasing through CP2 and Ediff. Interestingly, myeloid-affiliated genes such as Mpo also show this pattern (PU.1 seems to increase in entropy only in the late commitment CP1 population). Also of note is the fact that Gata2 displays the opposite behaviour to the other referred erythroid genes, decreasing in entropy in CP1 to then increase again in CP2 and Ediff.

## 5. Discussion

**Figure 3.**
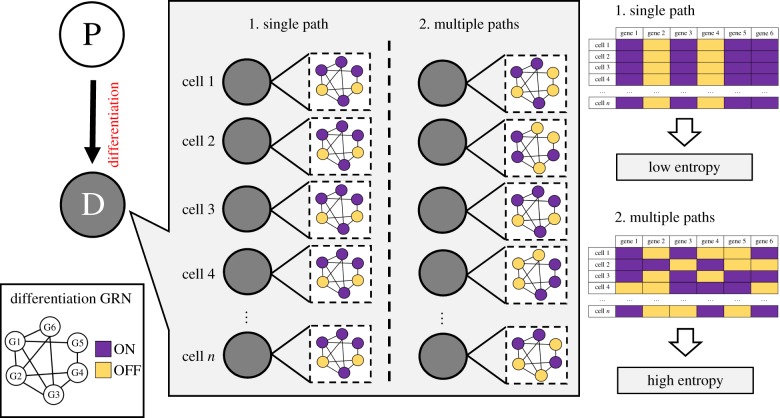
Increased binary Shannon entropy in post-commitment cell populations is consistent with multiple paths into the committed state and the coexistence of different states of a differentiation gene regulatory network (GRN). P, progenitor cells; D, differentiated cells. G1 to G6, gene 1 to gene 6 of a hypothetical differentiation GRN. Purple, gene is active (ON); orange, gene is inactive (OFF).

The interpretation of these results calls for a more careful interpretation of the entropy values and what they may signify in terms of the underlying biology of differentiation (figure 3). Mathematically, a gene has maximum entropy for a given population when half the cells of that population express the gene and the other half does not. High entropy just after a decision point, however, would be, naively, contrary to a more deterministic picture where, in order for a cell to progress to a more differentiated state, a set of key regulators would be required to be active and, likewise, key regulators of other lineages that could act as antagonists would need to be repressed. If this assumption was correct, we would expect the entropies of those key regulator genes to be low after a branching point such as the MPP to CMP/CLP transition, since they would be expected to be either always present or always absent in all post-commitment cells. Since cells can display a high level of heterogeneity in expression of key regulators even after commitment has occurred, this deterministic view is most probably not entirely accurate. These observations suggest that commitment into a more differentiated compartment could thus occur through multiple pathways, each representative of a different substate of the differentiation GRN. Higher values of entropy would then be caused by the different expression profiles of these GRN substates when more than one substate is present in the population.

Our results are consistent with the notion that entropy, as a measure of gene expression disorder, highlights the heterogeneous nature of cell fate decisions through multiple pathways defined by different GRN configurations. In the first analysed dataset, we observed that entropy increases after the MPP branching point, with both CMP and CLP populations showing significantly higher entropy values than that of MPP. We further expanded on this observation by analysing a second dataset which sampled populations of the EML cell line, allowing the capture of cellular states immediately before and after the erythroid commitment boundary. As before, we observed an increase in entropy immediately after commitment, from the SR to the CP1 population, consistent with our previous results. Furthermore, we explored the entropy values for single genes and observed this SR-to-CP1 increase for known erythroid regulators (e.g. Gata1, Klf1 and Fog1) as well as some myeloid regulators (e.g. Mpo) (figure 2*c*; electronic supplementary material, figure S1). Interestingly, Gata2 shows the opposite trend, with entropy decreasing to zero in the CP1 population, suggesting that for some regulators there is more stringent regulation leading to all cells of the committed population showing the same expression profile (in this case, all cells express Gata2). This result is consistent with previous predictions that Gata2 sets two regulatory modes in SR cells [5]: a restrictive mode when not expressed, effectively blocking commitment, and a permissive mode when expressed, allowing commitment to occur through different combinations of other regulators in the network.

There are still a number of potential caveats and unresolved questions that require further discussion. An important point is that in both datasets the gene set was chosen *a priori* and thus results are, by definition, biased. In other words, the entropy behaviour we observe is dependent on the set of genes under analysis. In both systems analysed here, gene selection was informed by potential relevance for the differentiation process, which in principle allows the entropy values to be informative in that context.

Another question regards the biological interpretation of high entropy values. In the context of the data, and in light of the work of others, we assume the existence of multiple entries into a committed or more differentiated state, in which case the interpretation of high entropy is the presence of disorder in the differentiation network, as given by that snapshot of the population (figure 3). Our interpretation of the temporary entropy increase due to the availability of several pathways to the next stage has the implicit assumption that, in this case, the choice among these is driven by internal noise. An additional possibility is that the decision is effected via an external signal as is suggested in [12]. In this work the authors make an analogy with chemistry principles and propose the existence of a ‘transition state’, heterogeneous at the population level, where individual cells exhibit different transcriptional profiles resulting in interconvertible substates of a differentiation gene expression network. The main difference between this transition state and what we consider to be the committed state is the fact that in the latter, and in virtue of the experimental data upon which we based our analysis, we do not consider the existence of a reversion probability from each of the subnetworks to a ‘pre-commitment’ configuration.

An alternative explanation, however, could be that high entropy comes from a gene that is not actively regulated, for instance, because it is not important for that population, in which case we would expect a 50/50 presence at any given moment for that population. This is very unlikely if we assume that, in order to save energy resources, a cell will most likely not express a gene until it has to do so [13]. In principle, high entropy genes could also be those with cyclic behaviour, e.g. a cell cycle gene. However, such genes are not included in our analysis.

Calculating joint entropies for more than one gene or mutual information values for small sets of genes allows us to distinguish potentially spurious high entropy values from cases where high entropies are the result of some degree of coordination between genes.

In the first part of our results, we followed the more classical description of the haematopoietic branching tree (figure 1*a*). It should be noted however that this is not a consensual description and multiple versions have been put forward based on different types of data [14]. Guo *et al.* suggest that their results support an alternative architecture where lymphomyeloid lineage commitment may happen upstream of the CLP/CMP separation [15–17]. In particular, through network inference methods and further validation experiments, they detected signs of coordinated MegE transcriptional priming in haematopoietic stem cells. Using the same set of 179 regulators, our entropy estimates still suggest increased activity at the CLP/CMP bifurcation.

From the point of view of the data themselves, we deliberately use only the binary information of whether gene activity is present or absent. A second aspect of the data is the continuous distribution of values when the gene is active, for which we are currently developing analysis protocols. From the biological point of view, we can say that in this paper we assume a ‘digital’ approach to gene expression where we consider all or nothing effects (the gene is either on or off). This may be a more adequate approximation to some genes than others, where ‘analogue’ regulation by fine-tuning expression levels may be more relevant. The digital and analogue views are also not mutually exclusive and a more careful exploration of the mechanistic basis and biological function of these two modes would greatly benefit the community [18,19].

**Figure 4.**
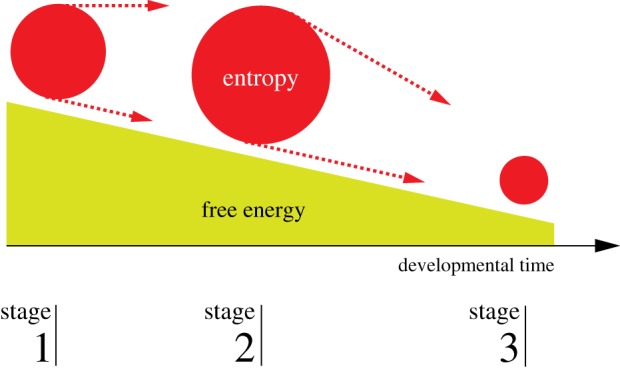
Schematic picture of the lineage development of entropies (red) through an intermediate stage. Also shown are the free energies (green) according to the Waddington metaphor [24]. The sizes of the red circles represent the amount of entropy at the different stages. The free energy is here shown as monotonically decreasing, which need not always be the case.

Related work includes [20], where it is argued in general terms that cell population entropy is positively related to developmental potency. In [21] one also investigates the hypothesis that entropy is monotonically decreasing during differentiation. The authors develop a Fokker–Planck-type model for the expression of a single gene, Sca1, from which they predict a probability density. They compute a differentiation potential which they find to continuously decrease and conclude that the initial density is close to the maximum entropy distribution. In [22], the signalling entropy [23] is computed for single-cell expression measurements during stem cell differentiation. The main difference from our analysis is in the computation of the entropy. The signalling entropy is extracted from a known protein–protein network whose edges are weighted by the single-cell expression data. This gives rise to a random walk on the network from which entropies are extracted. In contrast to this, our analysis uses the raw expression data directly to compute the entropy of the expression distribution, without the intermediate step of a network. Their results differ from ours as they exhibit a monotonic decrease throughout differentiation. All these previous studies conclude that both the entropy and a second variable, called free energy or developmental potency, are decreasing continuously during the differentiation process. Our analysis shows that the behaviour of the entropy is different from what is expected from these models. In figure 4, we show a schematic of the development of entropy and free energy during development. The size of the red circle indicates the first increasing and then decreasing entropy while the cell is on a free-energy slope towards commitment. Ideally one would compute the free energy from the data. But that requires a model for the internal energy for which one needs the interactions between all the participating genes together with model parameters, neither of which are known. Furthermore, with commonly used dynamic models, e.g. Hill equations, there is no energy function correspondence.

In [25], a similar entropy analysis was done using a different single-cell dataset. A non-monotonic decrease towards differentiation was found. However, the entropy estimation method does not take into account the dependency on the number of bins the data are discretized into, which we found to be significant—hence our choice to distinguish between on and off values only. Also, in [25] no comment is made on the statistical accuracy of estimating *N*/2 probabilities from the measurement of *N* cells. Given the known statistical limitation of a probability distribution estimate from very sparse data, as is the case in [25], we hesitate to make more detailed comparisons with our study.

The often repeated interpretation of (supposed) high entropy in the stem cell stage is that a cell is maximally noncommittal with respect to its identity in a differentiated stage. However, there might be a trade-off between high entropy, which involves expression of about half the genes but allows for a non-committal starting position, versus low expression, which is energetically cheaper but does not prepare for various different pathways to enter. In, for example, [26], nonlinear dynamic models of differentiating cells are presented, which can be considered to be a complementary approach to ours, where we in contrast present experimental data and a non-parametric analysis in terms of entropy.

### 5.1. Concluding remarks

In this study, we have found that the Shannon entropy is not a decreasing function of developmental pseudo-time, as predicted by others in the field, but instead it increases towards the point of differentiation before decreasing again. This behaviour was interpreted as different combinations of regulator activity, suggesting the presence of multiple configurations of the differentiation network as a result of multiple entry points into the committed state.

Assuming that the interpretation of increased entropy during commitment transitions is correct, a practical application of entropy measurements along a differentiation trajectory would be to measure the entropy in time series or pseudo-time series [27] from static gene expression data to obtain a signal for where crucial changes in development take place. This would allow narrowing in on important developmental transitions independently of surface marker classification of cellular populations.

## Supplementary Material

Figure S1:

## Data accessibility

The underlying data are openly available from the cited resources.
