# Supplementary material for: Haematopoietic stem cells: entropic landscapes of differentiation
Source: Interface Focus. 2018 Oct 19;8(6):20180040. doi: 10.1098/rsfs.2018.0040 (PMC6227807; doi:10.1098/rsfs.2018.0040)
Supplement: Figure S1: [file rsfs20180040supp1.pdf]

Supplementary Information S1

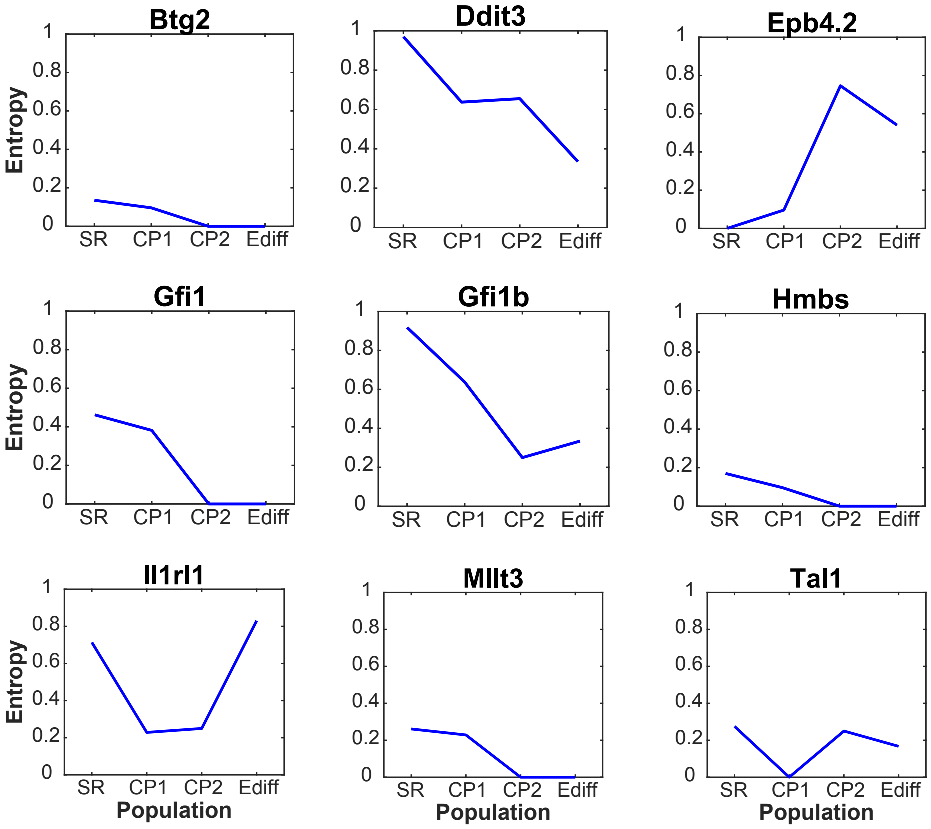

Figure S1: Binary Shannon entropy for individual genes quantified in different populations of the EML cell line as described in Figure 2 in the main text. SR - self-renewing cells; CP1 and CP2 are, respectively, early and late committed progenitors; Ediff - Erythroid-differentiated cells.
